# Comparison of Three Real-Time PCR Assays Targeting the SSU rRNA Gene, the COWP Gene and the DnaJ-Like Protein Gene for the Diagnosis of *Cryptosporidium* spp. in Stool Samples

**DOI:** 10.3390/pathogens10091131

**Published:** 2021-09-02

**Authors:** Felix Weinreich, Andreas Hahn, Kirsten Alexandra Eberhardt, Torsten Feldt, Fred Stephen Sarfo, Veronica Di Cristanziano, Hagen Frickmann, Ulrike Loderstädt

**Affiliations:** 1 Department of Microbiology and Hospital Hygiene, Bundeswehr Hospital Hamburg, 20359 Hamburg, Germany; felixweinreich@bundeswehr.org (F.W.); frickmann@bnitm.de (H.F.); 2 Department of Medical Microbiology, Virology and Hygiene, University Medicine Rostock, 18057 Rostock, Germany; andreas.hahn@uni-rostock.de; 3 Institute of Transfusion Medicine, University Medical Center Hamburg-Eppendorf, 20251 Hamburg, Germany; ki.eberhardt@uke.de; 4 Department of Tropical Medicine, Bernhard Nocht Institute for Tropical Medicine, 20359 Hamburg, Germany; 5 Department of Gastroenterology, Hepatology and Infectious Diseases, University Medical Center Düsseldorf, 40225 Düsseldorf, Germany; Torsten.Feldt@med.uni-duesseldorf.de; 6 Department of Medicine, Kwame Nkrumah University of Science and Technology, Kumasi 00233, Ghana; fsarfo.chs@knust.edu.gh; 7 Institute of Virology, Faculty of Medicine and University Hospital of Cologne, University of Cologne, 50935 Cologne, Germany; veronica.di-cristanziano@uk-koeln.de; 8 Department of Hospital Hygiene & Infectious Diseases, University Medicine Göttingen, 37075 Göttingen, Germany

**Keywords:** *Cryptosporidium* spp., real-time PCR, test comparison, latent class analysis, sensitivity, specificity, diagnostic accuracy

## Abstract

As qualified microscopy of enteric parasitoses as defined by high diagnostic accuracy is difficult to maintain in non-endemic areas due to scarce opportunities for practicing with positive sample materials, molecular diagnostic options provide less investigator-dependent alternatives. Here, we compared three molecular targets for the real-time PCR-based detection of *Cryptosporidium* spp. From a population of 1000 individuals comprising both Ghanaian HIV (human immunodeficiency virus) patients and military returnees after deployment in the tropics, stool samples were assessed for *Cryptosporidium* spp. by real-time PCR targeting the small subunit ribosomal RNA (SSU rRNA) gene, the *Cryptosporidium* oocyst wall (COWP) gene, and the DnaJ-like protein gene (DnaJ), respectively. In declining order, sensitivity of 100% for the SSU rRNA gene PCR, 90.0% for the COWP PCR and 88.8% for the DnaJ PCR, respectively, as well as specificity of 99.6% for the COWP PCR and 96.9% for both the SSU rRNA gene PCR and the DnaJ PCR, respectively, were recorded. Substantial agreement (kappa value 0.663) between the three assays was observed. Further, an accuracy-adjusted *Cryptosporidium* spp. prevalence of 6.0% was calculated for the study population. In conclusion, none of the assessed real-time PCR assays were associated with perfect test accuracy. However, a combination of highly sensitive SSU rRNA gene PCR for screening purposes and more specific COWP PCR for confirmatory testing should allow reliable diagnosis of *Cryptosporidium* spp. in stool samples even in low prevalence settings.

## 1. Introduction

Human cryptosporidiosis, which typically affects immunocompromised patients, has traditionally been diagnosed with acid-fast staining and subsequent microscopical assessment [1,2,3,4]. However, skilful microscopy is difficult to maintain in non-endemic settings [5], resulting in limited diagnostic accuracy even in European reference centres [6]. There, molecular diagnostic approaches like real-time PCR are widely applicable as standard diagnostic procedures with a high degree of automatization in the meantime. Therefore, less investigator-dependent modern molecular diagnostic assays for the detection of *Cryptosporidium* spp. have been shown to be more reliable compared to microscopical or antigen-based diagnosis of human cryptosporidiosis in numerous studies [7,8,9,10,11,12,13,14,15,16,17,18,19,20,21,22,23,24,25,26,27,28,29,30,31,32,33,34,35,36,37,38,39,40,41,42,43,44,45,46,47,48,49,50,51,52,53,54,55,56,57,58,59,60,61,62,63,64,65,66,67,68,69,70,71,72,73,74,75,76,77]. The sensitivity of molecular assays targeting *Cryptosporidium* spp. is influenced by factors like the mode of nucleic acid extraction as well as by the stage of the life cycle of the parasite [78,79,80,81,82,83,84,85]. In contrast, attribution of etiological relevance of detected *Cryptosporidium* spp. DNA in individuals in high prevalence settings can be challenging [86,87,88,89,90]. Due to the partly inconsistent results regarding PCR accuracy [7,8,9,10,11,12,13,14,15,16,17,18,19,20,21,22,23,24,25,26,27,28,29,30,31,32,33,34,35,36,37,38,39,40,41,42,43,44,45,46,47,48,49,50,51,52,53,54,55,56,57,58,59,60,61,62,63,64,65,66,67,68,69,70,71,72,73,74,75,76,77], diagnostic standardization is ongoing.

Although this assessment does not address challenges of the molecular diagnosis of *Cryptosporidium* spp. in human samples or its medical interpretation, such as cycle stage dependence, optimization of nucleic acid extraction or attribution of etiological relevance of *Cryptosporidium* spp.-specific DNA-detections, it focuses on another aspect of diagnostic standardization: the choice of the molecular target structure by conducting a comparative head-to-head in vitro assessment with human sample materials.

In detail, the aim of the study presented here was to contribute to the standardization of *Cryptosporidium* spp.-specific real-time PCR assays by a comparative evaluation of three commonly chosen target genes, namely the small subunit ribosomal RNA (SSU rRNA) gene, the *Cryptosporidium* oocyst wall (COWP) gene, and the DnaJ-like protein gene (DnaJ), respectively [48,51,91,92,93]. Those target sequences were chosen because they have been frequently applied in *Cryptosporidium* spp.-specific molecular assays in the past [7,8,9,10,11,12,13,14,15,16,17,18,19,20,21,22,23,24,25,26,27,28,29,30,31,32,33,34,35,36,37,38,39,40,41,42,43,44,45,46,47,48,49,50,51,52,53,54,55,56,57,58,59,60,61,62,63,64,65,66,67,68,69,70,91,92,93] and so the comparative assessment of assays targeting them might be of interest for many laboratories and assay producers. The assessment was performed as a test comparison without a reference standard with 1000 residual DNA aliquots from stool samples with a high pretest-probability of being positive for *Cryptosporidium* spp. DNA [94] applying latent class analysis (LCA) [95,96].

## 2. Materials and Methods

### 2.1. Study Population

Acquired from Ghanaian HIV patients (*n* = 905) [94,97,98,99] and German soldiers after returning from tropical deployments (*n* = 95) [98], a total of 1000 residual nucleic acid extractions from stool samples were included in the assessments. All residual stool samples were collected between 7 and 14 years prior to the test comparisons for diagnostic purposes and stored frozen at −80 °C. Thereby, microscopic results were not available, so the test comparisons were performed without a reference standard. In line with the ethical clearance obtained for the test comparisons, patient-specific data like age, sex or clinical history could not be presented, which is an admitted violation of the STARD (Standards for Reporting Diagnostic Accuracy) criteria [100].

### 2.2. Nucleic Acid Extraction and Real-Time PCR Assays

Nucleic acid extraction was performed by applying the QIAamp stool DNA mini kit (Qiagen, Hilden, Germany) as described by the manufacturer. The eluates were stored at −80 °C prior to the PCR analysis. Three real-time in house PCR assays for *Cryptosporidium* spp. targeting a 159-base pair sequence of the small subunit ribosomal RNA (SSU rRNA) gene, a 151-base pair sequence of the *Cryptosporidium* oocyst wall (COWP) gene, and a 138-base pair sequence of the DnaJ-like protein gene (DnaJ), respectively [48,51,91,92,93], were performed with all samples on magnetic induction cyclers (MIC, Bio Molecular Systems Ltd., London, UK). The applied oligonucleotides are shown in Table 1.

The reaction mix of each assay comprised the HotStarTaq Mastermix (Qiagen, Hilden, Germany) and a final Mg^2+^ concentration of 5 mM. For the different assays, the concentrations were 300 nM for the primers and 200 nM for the probe in the DnaJ-like protein gene-specific assay, 300 nM for the primers and 25 nM for the probe in the COWP gene-specific assay as well as 250 nM for the primers and 300 nM for the probe in the SSU rRNA gene-specific assay, respectively. The PCR reactions were run in 20 µL volumes including 2 µL residual DNA eluate. Each PCR run included a negative control based on PCR-grade water and a positive control based on a plasmid with inserted *Cryptosporidium* spp. sequences according to the NCBI GenBank accession numbers AY458612, AF248743, and AF188110, respectively, in a pEX-A128 vector backbone (see Table A1 for sequence details). Sample inhibition was assessed by applying a Phocid herpes virus DNA-specific real-time PCR as described elsewhere [101]. The limits of detection of the three real-time PCR assays were calculated based on a dilution series of the positive control plasmid applying the software SciencePrimer.com (http://scienceprimer.com/copy-number-calculator-for-realtime-pcr, last accessed on 13 July 2021). Limits of detection less than 10 copies per µL eluate were recorded for all real-time PCR assays. In detail, a limit of detection of 7.7 target genes per µL eluate was recorded based on the dilution series for all assays assessed. The PCR reaction profile was as follows: Initial heating to 95 °C for 15 min followed by 45 cycles of denaturation for 15 s at 95 °C and annealing as well as amplification for 60 s at 60 °C with subsequent final cooling to 40 °C for 20 s.

### 2.3. Exclusion Criteria and Statistical Assessment

Inhibited samples as indicated by the inhibition control PCR [101] were excluded from the analyses. Applying latent class analysis (LCA) [95,96], sensitivity and specificity of each real-time PCR assay as well as accuracy-adjusted prevalence of the study population were estimated. Fleiss’ kappa for the agreement of the different assays was calculated by applying the categories as previously described [102]. In addition, a comparison of the recorded cycle threshold (*Ct*) values was conducted. The software Stata/IC 15.1 for Mac 64-bit Intel (College Station, TX, USA) was used for the calculations.

## 3. Results

### 3.1. Agreement Kappa between the Real-Time PCR Assays, LCA-Based Calculation of Sensitivity as Well as Specificity of the Assays, and Accuracy-Adjusted Prevalence in the Study Population

From a total of 1000 samples, 33 had to be excluded from further assessment because of recorded PCR inhibition. Overall, two inhibited samples were from the subpopulation of the 95 German soldiers, and the other 31 were from the 905 Ghanaian HIV patients. Within the remaining 967 samples, 56 (5.79%) positive PCR signals were recorded in COWP PCR, 80 (8.27%) in DnaJ PCR and 86 (8.89%) in SSU rRNA gene PCR. Of note, all positive results were recorded from samples from Ghanaian HIV patients. Thereby, agreement between the three compared *Cryptosporidium* spp.-specific real-time PCR assays was substantially in line with the interpretation standards as suggested by Landis and Koch [102] with a Fleiss’ kappa value of 0.663. As calculated by applying LCA, the test accuracy-adjusted *Cryptosporidium* spp. prevalence within the assessed study population was 6.0% (Table 2).

Focusing on sensitivity as calculated with LCA, optimum sensitivity of 100% was recorded for SSU rRNA gene PCR followed by COWP PCR with 90.0% and DnaJ PCR with 88.8%, respectively. Regarding specificity, in contrast, COWP PCR scored best with 99.6%, while a lower specificity of 96.9% was calculated with LCA for both DnaJ PCR and SSU rRNA PCR (Table 2).

When focusing on the matches and mismatches between the different PCR assays, all three PCRs were positive in 47 samples and all three PCRs were negative in 851 cases. When directly comparing the positive results between the individual assays, the number of mismatches ranged between 3 and 33. In comparison, the numbers of matches between different assays ranged from 47 to 86 for positive results and from 854 to 911 for negative results. Details are provided in Table 3.

### 3.2. Comparison of the Cycle Threshold (Ct) Values between the Assays

Comparing the recorded cycle threshold values, highest mean and median Ct values were recorded for DnaJ PCR (Table 4), for which the lowest sensitivity was also calculated (Table 2). The mean and median Ct values of COWP PCR and SSU rRNA gene PCR, in contrast, were virtually identical. Even the difference between DnaJ PCR and COWP PCR or SSU rRNA gene PCR was quite low, ranging between just 1 and 2 Ct steps. The standard deviation ranges of the Ct values of all compared PCRs were also quite similar, varying between 3.84 and 4.87 (Table 4).

## 4. Discussion

The study was performed to comparably assess the diagnostic accuracy of three real-time PCRs targeting *Cryptosporidium* spp. based on three target genes, i.e., the small subunit ribosomal RNA (SSU rRNA) gene, the *Cryptosporidium* oocyst wall (COWP) gene, and the DnaJ-like protein gene (DnaJ), respectively [48,51,91,92,93], without a reference standard applying latent class analysis (LCA). Interestingly, substantial agreement [102] with a kappa value of 0.663 between the diagnostic assays indeed confirmed a common targeted meta-structure, but is yet far away from a perfect matching. In the LCA-based estimation indicating sensitivity ranging from 88.8% to 100% and specificity ranging from 96.9% to 99.6%, *Cryptosporidium* spp. was the common meta-structure addressed by the three assays with specificity for three different target sequences.

Close-to-perfect specificity (>99%) could be shown for the COWP PCR assay only, while calculated specificity for the SSU rRNA gene assay and the DnaJ PCR assay were still acceptable, but nevertheless imperfect, with values >95%. As specificity depends on the conservation level of the target sequence, limited numbers of available reference sequences, in particular for the DnaJ-like protein gene [91], imply uncertainty regarding their diagnostic reliability. Accordingly, in-vitro evaluations like the one described here are unavoidable. Regarding sensitivity, a close-to-perfect value of virtually 100% was recorded for the SSU rRNA gene PCR only, while sensitivity values of 90% as estimated for the COWP PCR and even slightly below 90% as estimated for the DnaJ PCR have to be considered as suboptimal for diagnostic purposes. Although previous assessments suggested even worse sensitivity of about 50% for microscopy-based identification of *Cryptosporidium* spp. in human stool samples [7], a situation with one out of ten samples positive for *Cryptosporidium* spp.-specific DNA going undetected still leaves room for improvement.

Of note, a total of 33/1000 samples (3.3%) had to be excluded from the assessment due to inhibition of the PCR reaction. Thereby, a higher proportion of inhibition was recorded in the samples collected under resource-limited tropical conditions compared to the samples collected under standardized conditions from the assessed German soldiers. Among the latter, the inhibition rate was close to 2%, which matched the expectations from a previous assessment [103] as well as the authors’ experience from the diagnostic routine. Considering the abovementioned sensitivity limits of microscopy as compared to real time PCR [7], the sensitivity benefit due to real-time PCR is still obvious.

In summary, none of the assessed assays showed optimum accuracy for the detection of the targeted meta-structure *Cryptosporidium* spp. Due to their imperfect specificity, SSU rRNA gene PCR and DnaJ PCR results should be confirmed by COWP PCR showing high specificity, while the highly sensitive SSU rRNA gene PCR is an interesting candidate for an initial screening. However, one has to keep in mind that the reduced specificity can result in deleterious consequences for the positive predictive value in case of screenings in populations with a low pretest probability due to a low prevalence in line with Bayes’ theorem [95]. Therefore, confirmatory testing applying the more specific COWP PCR is advisable in case of screening assessments in low prevalence settings. A likely combination could comprise the SSU rRNA gene PCR as a highly sensitive screening approach in order not to overlook infections as well as COWP PCR-based confirmatory testing for surveillance in low prevalence settings. When applying this combination of real-time PCR assays, a possible case of a *Cryptosporidium* spp. infection would be defined by a solitary positive SSU rRNA gene PCR, while a confirmed case would demand confirmation by a positive result in COWP PCR as well. The associated additional effort will be negligible, because the identical run conditions of both assays allow their application in single-tube or multi-tube multiplexing approaches, if the availability of the required technical equipment and trained personnel can be considered as guaranteed.

Interestingly, the lower sensitivity of COWP PCR compared to SSU rRNA gene PCR cannot be explained by cycle threshold value differences. On the contrary, the recorded cycle threshold values of both assays were quite similar. Although the SSU rRNA gene is a multicopy target, which potentially accounts for slightly better sensitivity, the low number of genomic repeats may explain the recorded similar Ct values. Hypothetically, the observed test characteristics were affected by the performed standard nucleic acid extraction with the column-based QIAamp DNA stool mini kit (Qiagen, Hilden, Germany), because better DNA yields in the case of harsher bead-beating-based extraction schemes have been described [78,79,80,81]. However, such a hypothetically reduced target DNA yield should have influenced all assessed real-time PCR assays in the same direction, although stochastic amplification in the limit-of-detection range may be considered.

As expected for immunocompromised individuals from tropical settings [1,2,3], a considerable accuracy-adjusted prevalence of 6.0% could be calculated by LCA for the Ghanaian HIV patients. Well in line with a previous surveillance study [104], *Cryptosporidium* spp. DNA was absent in stool samples of German military returnees from tropical deployments. Apart from small outbreaks at tropical deployment sites as reported previously from the European Union Training Mission (EUTM) in Mali [105], *Cryptosporidium* spp. was virtually absent in the gut of German soldiers after deployment [106].

This study has a number of limitations. First of all, lacking microscopic results for most residual samples made it necessary to perform a test comparison without a reference standard applying LCA. Second, a relatively low proportion of positive samples in spite of a high pretest probability in the assessed sample collection resulted in broad 95%-confidence intervals. Third, funding limits did not allow sequencing of PCR amplicons, so individual decisions on whether a discrepancy was due to sensitivity or specificity issues was impossible and test accuracy had to be estimated based on mathematical paradigms alone. Fourth, the wide age range of the assessed sample sets might have influenced the results. As, however, sample quality was the same for all compared real-time PCR assays, this limitation more likely had an impact on the prevalence estimations rather than on the assay comparison itself.

## 5. Conclusions

Imperfect test accuracy was recorded for all real-time PCR assays assessed. However, optimum sensitivity close to 100% for the detection of *Cryptosporidium* spp. could be recorded for the SSU rRNA gene PCR and excellent specificity of 99.6% for the COWP PCR. If highly sensitive SSU rRNA gene PCR is applied for screening purposes and more specific COWP PCR for confirmation testing, reliable screening results should be possible even in the case of application in low prevalence settings. The results of this study may be of interest for diagnostic microbiologists who clinically interpret the results of *Cryptosporidium* spp. PCRs based on the addressed target sequences. To the authors’ best knowledge, a respective direct head-to-head comparison of such frequently applied *Cryptosporidium* spp.-specific target sequences from the diagnostic routine has not been provided so far.

Of course, the assessment addressed the choice of the target sequence only. Other topics like the further standardization of the nucleic acid extraction [78,79,80,81,82] and of the clinical interpretation of positive real-time PCR signals [86,87,88,89,90] still remain unsolved and require additional investigations. Further, it is also advisable to confirm the results obtained in this assessment with a study design in line with the abovementioned STARD criteria [100].

## Figures and Tables

**Table 1:** Applied oligonucleotides of the compared *Cryptosporidium* spp.-specific real-time PCR assays.

Forward Primer Name	Forward Primer Sequence	Reverse Primer Name	Reverse Primer Sequence	Probe Name	Probe Sequence
SSU rRNA gene PCR according to [51]					
JVAF	5′-ATGACGGGTAACGGGGAAT-3′	JVAR	5′-CCAATTACAAAACCAAAAAGTCC-3′	JVAP18S	5′-CY3-CGCGCCTGCTGCCTTCCTTAGATG-BHQ-2-3′
DnaJ-like protein gene according to [48]					
DnaJ F	5′-CGCTTCTCTAGCCTTTTCATGA-3′	DnaJ R	5′-CTTCACGTGTGTTTGCCAAT-3′	DnaJ P	5′-CY5-CCAATCACAGAATCATCAGAATCGACTGGTATC-BHQ-2-3′
COWP gene PCR according to [92]					
COWP P702 F	5′-CAAATTGATACCGTTTGTCCTTCTG-3′	COWP P702 R	5′-GGCATGTCGATTCTAATTCAGCT-3′	COWP P702 P	5′-ROX5-TGCCATACATTGTTGTCCTGACAAATTGAAT-BHQ-2-3′

**Table 2:** Agreement kappa between the compared real-time PCR assays as well as sensitivity, specificity and accuracy-adjusted prevalence as calculated with latent class analysis (LCA).

Assay	*n*	Positives (%)	Sensitivity (0.95 CI)	Specificity (0.95 CI)	Kappa (0.95 CI)
DnaJ PCR	967	80 (8.27)	0.888 (0.770, 0.949)	0.969 (0.955, 0.978)	0.663 (0.574, 0.744)
COWP PCR	967	56 (5.79)	0.900 (0.773, 0.960)	0.996 (0.989, 0.998)	
SSU rRNA gene PCR	967	86 (8.89)	1 (0, 1)	0.969 (0.956, 0.979)	
Prevalence (0.95 CI)	0.060 (0.047, 0.078)				

0.95 CI = 95%-confidence intervals. *n* = numbers.

**Table 3:** Cross-table detailing mismatches between the different PCR assays. Green = matching results. Red = mismatching results. Black = not filled in to avoid repetition.

		DnaJ PCR		COWP PCR		SSU rRNA Gene PCR	
		Negative	Positive	Negative	Positive	Negative	Positive
DnaJ PCR	negative	887	0				
	positive	0	80				
COWP PCR	negative	878	33	911	0		
	positive	9	47	0	56		
SSU rRNA gene PCR	negative	854	27	878	3	881	0
	positive	33	53	33	53	0	86

**Table 4:** Recorded cycle threshold (Ct) values of the real-time PCR assays.

Assay	*n*	Mean (SD)	Median (Min, Max)
DnaJ PCR	80	34.14 (4.87)	34.41 (22.12, 41.50)
COWP PCR	56	32.14 (3.84)	33.16 (22.30, 38.07)
SSU rRNA gene PCR	86	32.09 (4.31)	33.21 (20.38, 39.16)

*n* = numbers. SD = standard deviation. Min = minimum. Max = maximum.

## Data Availability

All relevant data are provided within the manuscript. Raw data can be provided on reasonable request.

## Appendix A

**Table A1:** **Sequence inserts for the positive control plasmid**.

Positive Control Insert Based on *Cryptosporidium* spp. Sequences According to the NCBI GenBank Accession Numbers AY458612, AF248743 and AF188110.
GAATTCTACCGTGGCAATGACGGGTAACGGGGAATTAGGGTTCGATTCCGGAGAGGGAGCCTGAGAAACGGCTACCACATCTAAGGAAGGCAGCAGGCGCGCAAATTACCCAATCCTAATACAGGGAGGTAGTGACAAGAAATAACAATACAGGACTTTTTGGTTTTGTAATTGGAATGAGTTAAGAATTCATTAATTCAACAAATTGATACCGTTTGTCCTTCTGGTTTTGTTGAAGAAGGAAATAGATGTGTTCAATATCTCCCTGCAAATAAAATCTGTCCTCCTGGATTCAATTTGTCAGGACAACAATGTATGGCACCAGAATCAGCTGAATTAGAATCGACATGCCCACCTAATTCGAATTCCTACGTCTAACTTCACGTGTGTTTGCCAATGCATATGAAGTTATAGGGATACCAGTCGATTCTGATGATTCTGTGATTGGTAAAAAGTATAGAAAGCTCTCATTATTGATCCACCCTGATAAGACAAGTCATGAAAAGGCTAGAGAAGCGTTTGAAATACGAATTC
